# The First International Congress of Road Safety in Mashhad, Iran

**DOI:** 10.5249/jivr.v4i2.264

**Published:** 2012-07

**Authors:** Ali Gorji

**Affiliations:** ^a,^Razavi Neuroscience Research Center, Mashhad, Iran.; ^*b,*^Shefa Neuroscience Research Center, Tehran, Iran.; ^*c,*^Institut für Physiologie I, Westfälische Wilhelms-Universität Münster, Münster, Germany.

According to estimates, road traffic accidents account for approximately 3000 deaths on a daily basis, and 50 million injuries and 1.2 million deaths each year.^1^ In low-income and middle-income countries, road traffic accidents account for 85% of deaths and 90% of annual disability.^2^ Casualties of this magnitude are a serious problem throughout the world and create a significant impact on society.^2^ Road traffic injuries are also the leading cause of disability-adjusted life years lost (DALYs), and contribute significantly to the burden of disease in developing countries.^3^ The World Health Organization (WHO) classifies road traffic injury as a major and neglected public health challenge requiring a concerted prevention effort.^2^ Deaths and injuries due to road traffic crashes are especially devastating in developing nations, with marked disparities between these nations and their industrialized counterparts. It is predicted that between 2000 and 2020, road traffic deaths will decline by about 30% in industrial countries, but will increase substantially in developing countries.^2^ Peden (2005) states that in the next 20 years, road traffic injuries have the potential to be the third leading contributor to the global burden of disease. It is therefore necessary that countries at every level take charge in road traffic injury prevention.^4^

Iran has one of the highest incidences of fatality rates due to road traffic crashes in the world.^5^ In Iran, road traffic crashes caused more than 27,000 deaths, 270,000 injuries and approximately 6 billion U.S. dollars in direct and indirect financial costs in 2006. This means that every two minutes, one person is injured in a road traffic crash in Iran. Furthermore, Iran has had one of the highest incidences of fatal road traffic injury in the world.^5^ In the last 20 years, more than half a million people were killed in traffic injuries in Iran. It has been shown that the DALYs of road traffic injuries in Iran are more than 1.07 million, ranking it the highest among all other diseases.^6^

To develop effective road safety programs in the region, establish and maintain regional road safety partnerships, and attract funds and resources, the First International Road Safety Congress was held at the Razavi Neuroscience Center in Razavi Hospital from March 8th-9th, 2012. The Razavi Neuroscience Center was established at Razavi Hospital in Mashhad; a non-governmental hospital built by the Astan Quds Razavi Foundation to train neurologists, neurosurgeons, radiologists, general practitioners, neuroscientists, and nurses in the region.

Twenty-five scientists from around the world (USA, Germany, England, Egypt, Norway, Sweden, Brazil, Australia, Switzerland, New Zealand, Malaysia, India and Iraq) participated in this congress, alongside many local clinicians and scientists. The World Health Organization, as well as the Shefa Neuroscience Research Center, the Sarah Jane Brain Foundation (located in New York, USA), the Sina Trauma and Surgery Research Center, the Police Disciplinary Forces of the Islamic Republic of Iran, and the Disaster and Emergency Management Department from the Ministry of Health & Medical Education were the official partners of this congress. Lectures presented different topics ranging from global road safety actions, children and road safety, as well as road safety research, infrastructure/management, and education.

The amount of information shared represented 80 various articles in the form of lectures and posters. In addition to the congress, an exhibition was held with more than 100 posters presented on prevention of road accidents by the Razavi and Shefa Neuroscience Research Centers. During the congress, the participants decided to set up a non-governmental organization on road safety in the region to enhance efforts on road safety strategies, focus on the prevention of serious injuries and fatal crashes in spite of human fallibility, and identify local road safety needs and priorities. This group is also interested in safety measures targeting pedestrians and non-motorized forms of transportation. Many road safety initiatives and practices can be implemented at low cost and within a short time frame. These actions should be shared with relevant ministries (transportation, health, education, communication) by this organization. More than 40 percent of the audience in this congress was younger than 30 years of age; training of these young scientists by well-known specialists in road safety raises hope for a significant improvement of the future of Iran’s road safety.
